# Notes on Operative Dentistry and Its Advancement

**Published:** 1883-12

**Authors:** George H. Perine

**Affiliations:** New York


### NOTES ON OPERATIVE DENTISTRY AND ITS ADVANCEMENT.

BY GEORGE H. PERINE, D. D. S., NEW YORK.

(Continued from page 594.)

It is estimated that the amount of gold used annually for filling teeth by the 13,000 dentists in the United States is valued at over $500,000, and more than $100,000 worth of other materials is employed for the same purpose.

In 1840, Dr. Westcott discovered the properties of cohesive gold foil, and Dr. Arthur advocated its use, and in 1857 published a treatise on the subject fully explaining his mode of using the foil, and describing the instruments necessary for working the same. Still later, heavy foils were introduced into practice, but were not generally adopted. In 1853 A. J. Watt patented sponge gold, which was strongly recommended for filling by Drs. C. W. Ballard, Blakesly, Dwinelle, and others. The last named in 1855 published a treatise on its use, and described the instruments requisite for the purpose.

Platinum was used for a time as a material for filling, and silver was extensively employed for the same purpose, but was at length discarded on account of its easy oxidation and lack of ductility. Amalgam, which is now rather extensively used in practice, was first made by M. Tarcan, and was introduced into the United States about 1833. For a time its use was strongly opposed by many leading dentists, and it became the subject of a lively controversy, the outgrowth of which was, in several instances, considerable bitter personal feeling between those interested. In 1848 Dr. E. Parmly published a paper entitled: *The Properties and Effects of Amalgam Examined*, which was the same year followed by an article from the pen of Dr. John Trenor entitled: *Observations on Amalgam, with Cases.*

In 1855 Dr. Elisha Townsend presented to the specialty a formula of Dr. Wm. M. Hunter's, consisting of four parts of silver to five parts of tin, which proved highly acceptable. Dr. H. G. Luther introduced, in 1860, a preparation which he called adamantican, which was a white filling, composed, as he claimed, of unoxidizable metals, and not likely to corrode. At the present time there are many preparations made by different persons, containing metals which combine so perfectly with each other as to produce the most satisfactory results.

Metallic filling, composed of metals fusable at a low temperature, and thus capable of being "cast" in the cavity, were introduced, in 1864, by Dr. B. Wood, but failed to become popular.

Tin foil was used as early as 1820, and still holds its place as a very satisfactory material, not only for temporary but permanent fillings also, when the abrasion is slight.

About 1848 gutta-percha began to be used as a temporary filling, and in 1849 Dr. A. Hill obtained a patent upon a preparation for filling, of which gutta-percha constituted the principal element, and which was known as "Hill's stopping." Osteoplastic, a filling of oxy-chloride of zinc, was offered to the profession in 1857, by Dr. James Pearson, since which time a number of preparations have been presented, not differing essentially from this, except in name. Dr. J. P. Michaels, in 1874, recommended caps of gold or mother of pearl, and of various sizes, which were to be pressed into the cavity which had been previously prepared, and filled with plastic stopping, ready to receive them. "Slayton's felt foil" a preparation which was patented in 1878. It is a metallic filling of fibrous character, which is obtained by reducing the metals to shreds. It is claimed by its inventor, Dr. N. B. Slayton, that it is more cohesive and pliable than any filling heretofore used.

*Plastics and Plastic Filling* is the title of a work of some consequence by Dr. J. F. Flagg, which appeared in 1881.

Before turning our attention to another subject, it will perhaps be as well for us to refer to some particular methods which have been employed for securing a better anchorage for the foil in peculiar cases; namely, the adoption of gold screws, which have been in use for many years. After their introduction into practice they were improved upon by Dr. Mack, who prepared instruments for adjusting them, and made the screws of different sizes.

*Irregularities of the teeth* have of late years received particular attention from the specialty, and the best methods of correcting them have been carefully considered. It is not necessary for us to enter into details regarding the correction of the dental defects above referred to, the methods and theories of practice in this respect being all laid down in the text books, which are numerous and varied. Among the appliances resorted to are inclined planes, ligatures, springs, etc.

In 1846, Dr. Tucker recommended rubber tubing for ligatures, and in 1857, Dr. Dwinelle employed and recommended the jack screw for the expansion of the jaw. Soft and hard rubber, gold and cast plates are principally employed for this purpose.

Dr. Joseph H. Foster recommended for some cases the spiral spring, and for the purpose of preventing the displacement of rubber ligatures gold screws were often brought into service by him.

In 1841, Dr. Solyman Brown wrote an essay upon the subject of regulating the teeth of children during the period of second dentition, and Dr. W. Dalrymple, in 1868-9, published accounts of several interesting cases of irregularities which he had succeeded in correcting, and upon the same subject Dr. J. N. Farrar has recenty published a series of valuable papers.

*Restoring teeth* is a department of operative dentistry in which great improvements have been made within the past half century. At the beginning of the nineteenth century, natural or animal teeth were engrafted upon roots by metallic or wood pivots. About 1820 porcelain pivot teeth began to be used. They were improved upon by Dr. Hudson, and others.

Dr. E. A. Bigelow obtained, in 1827, a patent upon a mode of engrafting artificial teeth with wood pivots and gold cylinders upon the roots.

Dr. J. S. Dodge, in 1844, recommended a pivot consisting of wood enclosing a metallic tube, which formed an opening for the escape of pus and gas.

In 1848, Dr. H. Lawrence introduced another method of pivoting. The artificial tooth was made with a hole entirely through it, into which a screw pivot passed, entering the root which had been previously prepared to receive it. During the following year he obtained a patent upon this method.

In 1849, Dr. F. H. Clark recommended a metallic pivot, made to fit a cylinder set in the root, through which it passed. The tooth could be easily removed for the purpose of cleansing.

In 1862, Dr. B. Wood presented a method for restoring the form and beauty of decayed and broken teeth. The appliances he proposed were caps of different sizes, intended to cover the entire crown of the tooth, made from tooth-body material of different shades, having grooves, slots, or platina pins baked in. These crowns to be engrafted upon the remains of teeth, with plastic filling.

Since 1862, various methods of attaching artificial crowns of gold or porcelain have been introduced by Drs. Foster, Richmond, Wetherbee, Bonwill and others, the latter gentleman having obtained a patent for his invention in 1881.

Dr. W. N. Morrison claims to have employed gold crowns as early as in 1869, but a patent for a similar method was, however, obtained by Dr. John B. Beers, of California, in 1873, whose claim to the origination of these crowns is generally believed to be well founded, as he had employed them as early as 1868.

In 1846, Dr. H. Crane issued the first Dental Register, in which all operations performed may be recorded, and a space where remarks relative to the same may be entered. Since the above date there have been several registers presented, one possessing an advantage over the other, yet each good in its way.

We will not attempt to enumerate here the almost countless, yet valuable contributions in the form of mechanical appliances and improved intruments, which have within the past fifty years been presented to the specialty, but in another department a list will be given of all such for which patents have been obtained.

